# Correlation of MRI-Lesion Targeted Biopsy vs. Systematic Biopsy Gleason Score with Final Pathological Gleason Score after Radical Prostatectomy

**DOI:** 10.3390/diagnostics11050882

**Published:** 2021-05-15

**Authors:** Mike Wenzel, Felix Preisser, Clarissa Wittler, Benedikt Hoeh, Peter J. Wild, Alexandra Tschäbunin, Boris Bodelle, Christoph Würnschimmel, Derya Tilki, Markus Graefen, Andreas Becker, Pierre I Karakiewicz, Felix K. H. Chun, Luis A Kluth, Jens Köllermann, Philipp Mandel

**Affiliations:** 1 Department of Urology, University Hospital Frankfurt, University Frankfurt, 60590 Frankfurt, Germany; felix.preisser@kgu.de (F.P.); Clarissa.wittler@kgu.de (C.W.); Benedikt.hoeh@kgu.de (B.H.); andreas.becker@kgu.de (A.B.); felix.chun@kgu.de (F.K.H.C.); luis.kluth@kgu.de (L.A.K.); philipp.mandel@kgu.de (P.M.); 2 Cancer Prognostics and Health Outcomes Unit, Division of Urology, University of Montreal Health Center, Montreal, QC H2X 3E4, Canada; c.wuernschimmel@gmail.com (C.W.); pierre.karakiewcz@umontreal.ca (P.I.K.); 3 Dr. Senckenberg Institute of Pathology, University Hospital Frankfurt, 60590 Frankfurt, Germany; peter.wild@kgu.de (P.J.W.); alexandra.tschaebunin@kgu.de (A.T.); jens.koellermann@kgu.de (J.K.); 4 Frankfurt Institute for Advanced Studies (FIAS), 60590 Frankfurt, Germany; 5 Wildlab, University Hospital Frankfurt MVZ GmbH, 60590 Frankfurt, Germany; 6 Department of Diagnostic and Interventional Radiology, University Hospital Frankfurt, 60590 Frankfurt, Germany; boris.bodelle@kgu.de; 7 Martini-Klinik Prostate Cancer Center, University Hospital Hamburg-Eppendorf, 20251 Hamburg, Germany; tilki@uke.de (D.T.); graefen@uke.de (M.G.); 8 Department of Urology, University Hospital Hamburg-Eppendorf, 20251 Hamburg, Germany

**Keywords:** fusion biopsy, systematic biopsy, concordance, upgrading, downgrading, prostate neoplasm

## Abstract

Background: The impact of MRI-lesion targeted (TB) and systematic biopsy (SB) Gleason score (GS) as a predictor for final pathological GS still remains unclear. Methods: All patients with TB + SB, and subsequent radical prostatectomy (RP) between 01/2014-12/2020 were analyzed. Rank correlation coefficient predicted concordance with pathological GS for patients’ TB and SB GS, as well as for the combined effect of SB + TB. Results: Of 159 eligible patients, 77% were biopsy naïve. For SB taken in addition to TB, a Spearman’s correlation of +0.33 was observed regarding final GS. Rates of concordance, upgrading, and downgrading were 37.1, 37.1 and 25.8%, respectively. For TB, a +0.52 correlation was computed regarding final GS. Rates of concordance, upgrading and downgrading for TB biopsy GS were 45.9, 33.3, and 20.8%, respectively. For the combination of SB + TB, a correlation of +0.59 was observed. Rates of concordance, upgrading and downgrading were 49.7, 15.1 and 35.2%, respectively. The combined effect of SB + TB resulted in a lower upgrading rate, relative to TB and SB (both *p* < 0.001), but a higher downgrading rate, relative to TB (*p* < 0.01). Conclusions: GS obtained from TB provided higher concordance and lower upgrading and downgrading rates, relative to SB GS with regard to final pathology. The combined effect of SB + TB led to the highest concordance rate and the lowest upgrading rate.

## 1. Introduction

Prostate cancer is still the most common cancer in men [1,2,3]. After diagnosis, histological prostate biopsy results are used for clinical decision-making with patients, aiming for potentially curative treatment in localized prostate cancer disease [4]. For clinical decision-making, surgical planning and counseling of patients with the possible need of adjuvant or salvage treatment, or for biochemical recurrence after radical prostatectomy, recommendations are mostly based on biopsy Gleason score (GS) [5]. Therefore, the biopsy GS should ideally be concordant with the pathologic GS results after radical prostatectomy to avoid over- or underestimating the disease. 

We recently characterized multiple small samples from the same radical prostatectomy at the genomic, transcriptomic, and proteomic levels including network-based integration of these multi-omics data [6]. Our results demonstrate the importance of detecting the so-called index tumor (with the highest GS) by a clever combination of biopsy approaches, as this is the only way to analyze and measure the biology of the disease as accurately as possible.

Within recent years, multiparametric magnetic-resonance imaging (MRI)-target prostate biopsy (TB) of suspicious lesions in MRI, in addition to a systematic 12-core biopsy (SB), has become frequently employed [7]. Multiple studies and meta-analyses investigated a higher concordance of biopsy GS in the combined effect of TB + SB compared to SB alone regarding final pathological GS [8,9,10,11,12,13,14]. However, in these studies an undeniable proportion of patients are affected by up- or downgrading after pathological results. The main limitation of those study may be the fact that different patient cohorts of TB (or TB + SB) vs. SB patients were compared. Ideally, biopsy GS results of TB and SB results should be compared within the same patient according to pathological GS, which has been investigated in fewer studies so far [15,16,17,18,19,20,21]. In consequence, uncertainty as to whether the prediction of pathological GS should be based on TB or SB results still remains.

We addressed this gap and relied on our institutional prostate biopsy and RP database to investigate the correlation of TB vs. SB GS within the same patient with pathologic GS after radical prostatectomy. We hypothesized that differences in predicting pathological GS may exist with regard to TB vs. SB. 

## 2. Materials and Methods

### 2.1. Study Population

The study was conducted in accordance with the Declaration of Helsinki. After approval of the local ethics committee (SUG-7-2020), all patients with prostate biopsy and subsequent radical prostatectomy after prostate cancer diagnosis between 01/2014 and 12/2020 at the Department of Urology, University Hospital Frankfurt, Germany, were identified. Exclusion criteria consisted of the performance of SB only without MRI-fusion TB (*n* = 99). Indications for prostate biopsies were ≥PIRADS 3 lesion in MRI (in accordance with the PIRADS-v2 classification) and/or suspicious digital rectal examination (DRE) and/or elevated PSA values. These selection criteria resulted in 159 eligible patients. 

### 2.2. Prostate Biopsy Approach and Performance

All biopsies were performed with a transrectal approach under antibiotic prophylaxis and periprostatic local anesthesia, as recommended and previously described [4,22]. For SB, 12-core biopsies with a length of 15–22 mm were taken (six biopsies per prostate lobe). TB was performed with a high-end ultrasound machine (HiVison, Hitachi Medical Systems, Tokyo, Japan). For TB, at least two cores were taken from each mpMRI lesion ≥PIRADS 3. In all included patients, both TB and SB were performed in combination. All biopsies were performed by five urologists with experience in prostate biopsies who performed both SB and TB. MRIs were initially screened by a board-certified radiologist. Biopsy Gleason score results of TB and SB were reported separately and the highest Gleason score of each patients’ TB and SB was used for analyses. Both biopsy and pathological Gleason score after RP were analyzed by experienced uropathologists (JK, PJW) and confirmed by an independent second uropathologist in accordance with the ISUP Consensus Conference 2014 grading system [23]. Upgrading and downgrading were defined as an upgrade/downgrade of at least one Gleason pattern. Moreover, significant upgrading/downgrading was defined as an upgrade/downgrade of at least two ISUP categories.

### 2.3. Statistical Analysis

Descriptive statistics included frequencies and proportions for categorical used variables. Medians and interquartile ranges (IQR) were reported for all used continuous variables. The Chi-square test was used to test for statistical significance in proportions’ differences. The t-test and Kruskal-Wallis test examined the statistical significance of distribution differences. 

The Spearman’s correlation coefficient was calculated for TB vs. SB and the combined effect of TB + SB. Moreover, concordance rates were calculated. Non-correlating results were classified as either upgrading or downgrading. All tests were two sided with a level of significance set at *p* < 0.05. R software environment for statistical computing and graphics (version 3.4.3, R foundation for statistical computing, Vienna, Austria) was used for all analyses.

## 3. Results

### 3.1. Descriptive Baseline Characteristics

Of all eligible 159 patients with TB and SB and subsequent RP, median age was 66 years (IQR 62–71) at time of biopsy (Table 1). Median PSA and prostate volume were 7.0 ng/mL (IQR 5.2–10.0) and 43 cc (IQR 35–60), respectively. The majority of patients were biopsy naïve (*n* = 122, 76.7%). Median number of cores taken at biopsy were 14 (IQR 14–15) and median number of positive cores were 5 (IQR 3–8). Moreover, median positive cores in TB and in SB were respectively 1 (IQR 1–2) and 3 (IQR 2–6). Of all patients, the majority harbored PIRADS 4 lesions on MRI (*n* = 68, 42.8%), followed by PIRADS 5 (*n* = 61, 38.4%) and PIRADS 3 (*n* = 23, 14.5%) in that order.

### 3.2. Correlation between GS in SB and Final Pathological GS after RP

**Figure 1:**
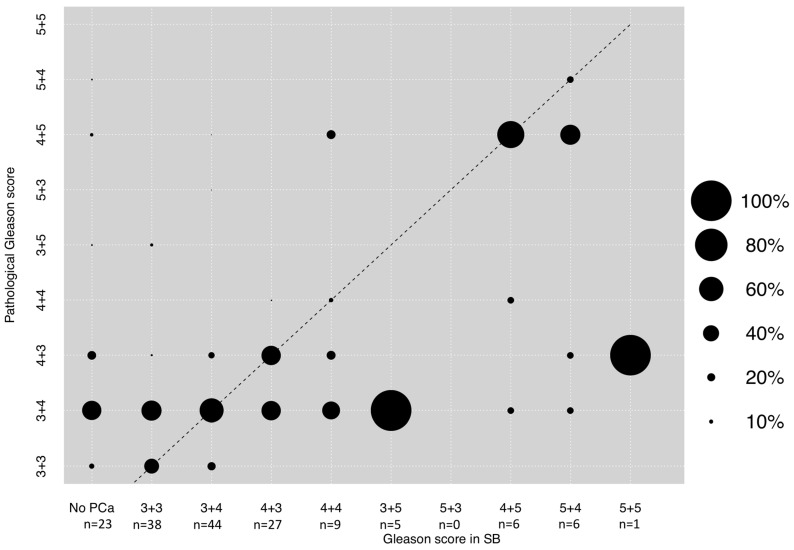
Dots depicting the concordance between the highest biopsy Gleason score in the systematic biopsy (SB) and final pathological Gleason score after radical prostatectomy. The Spearman’s correlation coefficient was + 0.33. The dotted line reflects the ideal correlation. Size of dots reflects percentages of patients as outlined in the legend. Abbreviations: PCa: Prostate cancer.

The majority of patients with SB in addition to TB exhibited GS 3 + 4 (*n* = 44, 27.7%), followed by 3 + 3 (*n* = 38, 23.9%) and 4 + 3 (*n* = 27, 17.0%), in that order. In those patients, prostate cancer was missed in 23 patients (14.5%) and the majority of 27 patients with Gleason 8-10 harbored GS 4 + 4 (*n* = 9, 5.7%), followed by 4 + 5 (*n* = 6, 3.8%), 5 + 4 (*n* = 6, 3.8%), 3 + 5 (*n* = 5, 3.1%), and 5 + 5 (*n* = 1, 0.6%). A Spearman’s correlation of +0.33 was observed according to final Gleason score pathology (Figure 1). The rates of concordance, upgrading and downgrading of SB GS were 37.1, 37.1, and 25.8%, respectively (Table 2). Significant upgrading and downgrading rates were 4.8% and 7.9%.

### 3.3. Correlation between GS in TB and Final Pathological GS after RP

**Figure 2:**
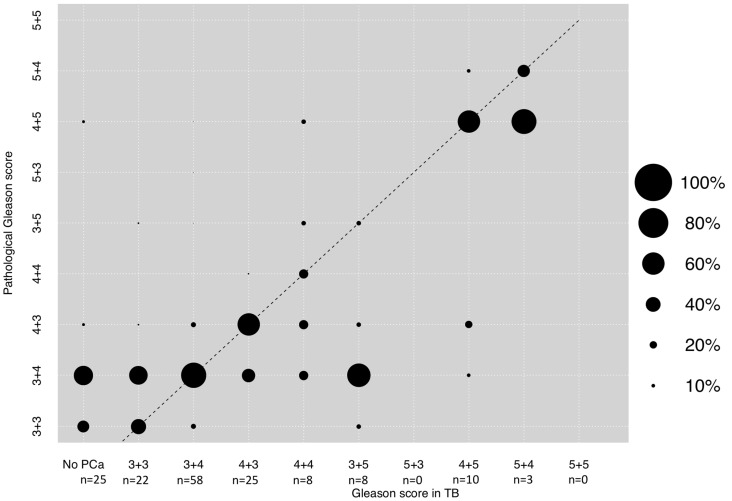
Dots depicting the concordance between biopsy Gleason score in the MRI-target lesion (TB) and final pathological Gleason score after radical prostatectomy. The Spearman’s correlation coefficient was + 0.52. The dotted line reflects the ideal correlation. Size of dots reflects percentages of patients as outlined in the legend. Abbreviations: PCa: Prostate cancer.

The majority of patients with TB exhibited GS 3 + 4 (*n* = 58, 36.5%), followed by Gleason 4 + 3 (*n* = 25, 15.7%) and missed prostate cancer detection in the MRI lesion (*n* = 25, 15.7%). GS 3 + 3 was detected in 22 patients (13.8%) and the majority of 29 patients with GS 8–10 harbored a 4 + 5 pattern (*n* = 10, 6.3%), followed by 4 + 4 (*n* = 8, 5.0%), 3 + 5 (*n* = 8, 5.0%), and 5 + 4 (*n* = 3, 1.9%). A Spearman’s correlation of + 0.52 was observed according to final GS pathology after radical prostatectomy (Figure 2). The rates of concordance, upgrading and downgrading of MRI-targeted lesion biopsy GS were 45.9%, 33.3%, and 20.8%, respectively (Table 2). Rates of significant upgrading and downgrading were 3.0% and 6.7%.

### 3.4. Correlation between GS in Combined SB + TB and Final Pathological GS after RP

**Figure 3:**
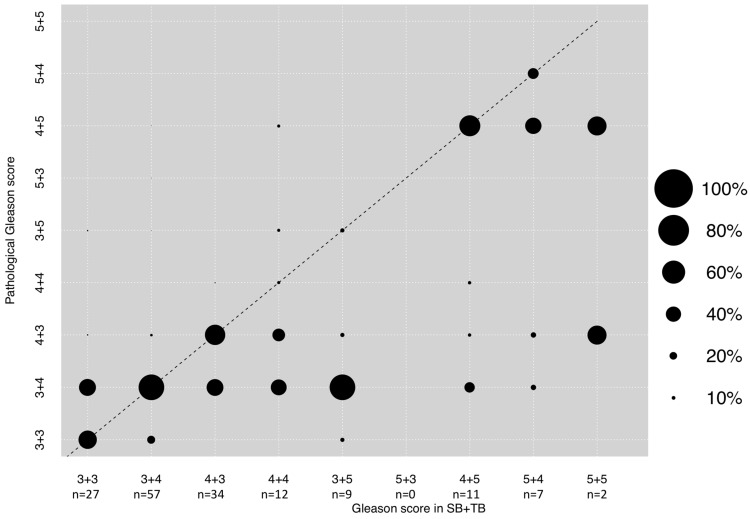
Dots depicting the concordance between the highest biopsy Gleason score regardless of MRI-lesion targeted (TB) or systematic biopsy (SB) and final pathological Gleason score after radical prostatectomy. The Spearman’s correlation coefficient was + 0.59. The dotted line reflects the ideal correlation. Size of dots reflects percentages of patients as outlined in the legend.

In the combined group of highest biopsy Gleason score in either the SB or TB, Gleason score 3 + 4 (*n* = 57, 35.8%) was the most frequent Gleason pattern, followed by 4 + 3 (*n* = 34, 21.4%) and 3 + 3 (*n* = 27, 17.0%), in that order. A Spearman’s correlation of +0.59 was computed according to correlation with final Gleason score pathology (Figure 3). The rates of concordance, upgrading and downgrading of SB + TB GS were 49.7, 15.1, and 35.2%, respectively. The combined effect of SB + TB resulted in a significantly lower upgrading rate, relative to TB and SB (both *p* < 0.001), but a higher downgrading rate, relative to TB (*p* < 0.01, Table 2). Significant upgrading and downgrading rates were 3.0% and 11.5%.

## 4. Discussion

We hypothesized that differences in predicting final pathological Gleason score may exist with regard to biopsy GS of SB and TB within the same patient. We tested this hypothesis in our institutional biopsy and radical prostatectomy database and arrived at several noteworthy findings. 

First, we observed important differences regarding biopsy GS in SB and final pathology GS. Specifically, SB in addition to TB was only moderately positively correlated with predicting final pathology (Spearman’s correlation + 0.33). Moreover, concordance rate was relatively low (37.1%), while conversely upgrading and downgrading rates were high (37.1% and 25.8%). These observations do not completely agree with previous publications, where SB and TB were not compared within the same patient. For example, Rührup et al. and Apfelbeck et al. reported a concordance of between 46.7%–48.9% with SB alone [8,11]. Conversely, the upgrading rate in the study by Apfelbeck et al. was invariably higher (46.7%). However, comparisons between these studies and our cohort are difficult due to different study designs. Comparing our results with studies performing and comparing SB vs. TB within the same patient, concordance also differed. For example, in the study by Diamand et al., the concordance rate for SB alone was 49.4% [18]. Moreover, in another study by Borkowetz et al., the rates of concordance and upgrading were 54 and 44% [19]. Furthermore, another report by Raipsarda et al. observed high concordance rates of 67% [24]. In consequence, compared to the current literature, the SB of our study showed lower concordance rates, but also lower upgrading rates. Since most studies relied on retrospective cohorts, further prospective trials and meta-analyses should ideally further investigate upgrading rates for better patient counseling prior to treatment decision of possible active treatment, so as not to underestimate the risk of upgrading.

Moreover, it is of note that within Gleason 8–10 patterns, a minority of patients with biopsy Gleason 4 + 4 in SB was upgraded and no patient with any Gleason 5 pattern (3 + 5, 5 + 3, 4 + 5, 5 + 4 or 5 + 5) was upgraded at all in our SB cohort. These observations are in an agreement with previously published literature. Specifically, Ploussard et al. found that upgrading mostly affects a change from low to intermediate risk prostate cancer [25]. Moreover, in large-scale epidemiological database study, Gansler et al. demonstrated that Gleason 8 (4 + 4, 3 + 5 and 5 + 3) is downgraded in 60% of cases, irrespectively of biopsy approach and used methodology [26].

Second, we also made important observations according to the correlation of TB Gleason score regarding final pathology after radical prostatectomy. More specifically, a Spearman’s correlation of + 0.52 was observed regarding final pathology, which indicates high correlation with final pathology. Moreover, concordance rates were invariably higher (45.9%), and upgrading (33.3%) and downgrading rates (20.8%) were substantially lower in the TB Gleason scores, relative to the SB Gleason scores, despite not reaching statistical significance. These observations may be linked to the index lesion, which may be identified through MRI and therefore led to higher concordance rates at RP [21,27]. Moreover, these observations are also in an agreement with previously published data about the concordance with final pathology of SB vs. TB Gleason score in separate patients. For example, a recently published meta-analysis included ten studies of which four studies included SB and TB results from the same patient and six studies included SB and TB patients from different cohorts [13]. Goel et al. found that upgrading rates are significantly higher in SB Gleason patterns, while concordance is significantly more frequent in TB Gleason patterns. These observations are particularly important since MRI-targeted biopsies are increasing within recent years due to improving quality, as well as easy and quick performance [28,29,30,31]. In consequence, whenever TB is performed and separate biopsy GS are available for TB and SB, clinical decision-making and patient counseling should rather be based on the findings of TB GS than on SB GS for predicting final pathology. However, the question remains whether TB is sufficient to predict true GS after radical prostatectomy alone or only in combination with SB.

Third, when results of TB and SB Gleason scores were combined, important observations were also made. Despite the fact that the Spearman’s correlation coefficient (+0.59) and concordance rates (49.7%) were the highest within all three examined groups, downgrading rates increased when TB and SB were combined (35.2%). The latter may be explained by the fact that the additional TB weighted the index tumor with the highest GS numerically more in biopsy pathology than was the case when GS after radical prostatectomy was surveyed. Similar observations were made in a study by Radtke et al. [21].

Conversely, upgrading rates were markedly low (15.1%). These findings are interesting when they are compared to our TB findings. Despite slight advantages for the combined use of SB and TB in terms of concordance (49.7 vs. 45.9%), upgrading (15.1 vs. 33.3%) and downgrading (35.2 vs. 20.8%), rates were markedly and significant different between SB + TB vs. TB alone. These observations give clinicians the opportunity to choose between a more conservative and more progressive estimation of final pathology. This means that reliance on the combined effect of SB and TB provides more robust and reliable information in terms of low risk of upgrading but high risk of downgrading, and vice versa for the effect of TB Gleason score alone. The findings regarding the combined effect of SB and TB are also in an agreement with the current literature. For example, in a study by Arsov et al., the combined effect of both techniques also showed the lowest rates of upgrading (29%), relative to SB (50%) and TB alone (40%) [32]. However, these rates differ to the rates of the current study and may be based on the fact of small sample size (*n* = 52). In another recently published study by Manceau et al., downgrading rates with the combined effect of TB and SB were 36% and very similar to the current study (35.2%) and emphasize the validity of our findings [33]. The fact that one third of all patients is downgraded is very important since these patients may have undergone active treatment, while they may have been candidates for less invasive treatments. Nonetheless, it is important to mention that, when significant upgrading and also significant downgrading rates were compared between all groups of SB, TB and SB + TB, no significant differences were observed.

Our study has several limitations and must be considered in the light of its retrospective design. In addition, the physicians who performed the biopsies were not blinded and performed both TB and SB. Moreover, SB was not blinded to MRI information and may have differed to SB, without MRI information prior to biopsy. Third, the results and lack of statistical significance may be affected by the limited sample size. Unfortunately, because of this limitation, subgroup analyses between biopsy-naive and repeat biopsy patients could not be calculated. Moreover, some of the results derived from multiple group comparisons. Finally, no information about an index lesion were available which could be compared to SB or TB results.

Overall, our study shows that the biopsy GS obtained from TB results has higher concordance and lower upgrading and downgrading rates compared with the SB GS with respect to final pathology. The combined effect of TB and SB resulted in the highest concordance rate and the lowest upgrading rate. Conversely, downgrading rates were highest when the results of TB and SB were combined.

## 5. Conclusions

GS obtained from TB provided higher concordance and lower upgrading and downgrading rates, relative to SB in addition to TB GS with regard to final pathology. The combined effect of SB + TB led to the highest concordance rate and the lowest upgrading rate. However, the highest rates of downgrading were observed. When significant upgrading or downgrading rates (at least two ISUP categories) were compared, no significant differences between all techniques were observed. 

## Figures and Tables

**Table 1:** Descriptive characteristics of 159 patients who underwent fusion biopsy and subsequent radical prostatectomy. Abbreviation: PSA: Prostate-specific antigen, MRI: Magnet resonance imaging, DRE: Digital rectal examination, PCa: Prostate cancer.

Variable		Overall *n* = 159
Age, Years	Median (IQR)	66 (62–71)
Prostate volume, cc	Median (IQR)	43 (35–60)
PSA, ng/mL	Median (IQR)	7.0 (5.2–10.0)
Cores taken at biopsy	Median (IQR)	14 (14–15)
Number of positive cores	Median (IQR)	5 (3–8)
Positive cores in MRI lesion	Median (IQR)	1 (1–2)
Positive cores out of lesion	Median (IQR)	3 (2–6)
DRE	non-suspicous	101 (63.5)
	suspicous	58 (36.5)
cT-stage	cT1	101 (63.5)
	cT2	56 (35.2)
	cT3-4	2 (1.3)
Prior biopsies	0	122 (76.7)
	1	31 (19.5)
	≥2	6 (3.8)
PIRADS lesion	PIRADS 3	23 (14.5)
	PIRADS 4	68 (42.8)
	PIRADS 5	61 (38.4)
	Unknown	7 (4.4)
Gleason score in lesion	No PCa	25 (15.7)
	3 + 3	22 (13.8)
	3 + 4	58 (36.5)
	4 + 3	25 (15.7)
	4 + 4	8 (5)
	3 + 5	8 (5)
	4 + 5	10 (6.3)
	5 + 4	3 (1.9)
	5 + 5	0 (0)
Gleason score out of lesion	No PCa	23 (14.5)
	3 + 3	38 (23.9)
	3 + 4	44 (27.7)
	4 + 3	27 (17)
	4 + 4	9 (5.7)
	3 + 5	5 (3.1)
	4 + 5	6 (3.8)
	5 + 4	6 (3.8)
	5 + 5	1 (0.6)
Pathological Gleason score	3 + 3	26 (16.4)
	3 + 4	80 (50.3)
	4 + 3	31 (19.5)
	4 + 4	3 (1.9)
	3 + 5	4 (2.5)
	5 + 3	1 (0.6)
	4 + 5	12 (7.5)
	5 + 4	2 (1.3)
	5 + 5	0 (0)
pT-stage	pT2	91 (57.2)
	≥pT3	68 (42.8)

**Table 2:** Concordance, upgrading and downgrading of biopsy Gleason score (GS) according to the highest biopsy GS regardless of the distinction between SB and TB, highest biopsy GS in TB and highest biopsy GS in SB.

	Highest GS regardless of SB or TB (A)	Highest GS in TB (B)	Highest GS in SB (C)	*p* Value A vs. B vs. C	*p* Value A vs. B	*p* Value A vs. C	*p* Value B vs. C
Concordance	79 (49.7%)	73 (45.9%)	59 (37.1%)	0.068	0.6	0.03	0.14
Upgrading	24 (15.1%)	53 (33.3%)	59 (37.1%)	<0.001	<0.001	<0.001	0.5
Downgrading	56 (35.2%)	33 (20.8%)	41 (25.8%)	0.01	<0.01	0.09	0.4
Significant upgrading	5 (3.0%)	5 (3.0%)	8 (4.8%)	0.6	1	0.6	0.6
Significant downgrading	19 (11.5%)	11 (6.7%)	13 (7.9%)	0.3	0.2	0.4	0.8

## Data Availability

Data will be made available on request for bona fide researchers.
